# Lipidomics and Transcriptomics Analyses Reveal Dietary Complex Plant Extracts Improve Lipid Composition of Back Fat in Sheep

**DOI:** 10.3390/ani15111645

**Published:** 2025-06-03

**Authors:** Hui Guo, Ruixue Nie, Tao Guo, Chang Gao, Jinju Mao, Yuchao Hu, Wenwen Wang, Na Liu, Xiaoping An, Jingwei Qi, Yuan Wang

**Affiliations:** 1 College of Animal Science, Inner Mongolia Agricultural University, Hohhot 010018, China; guohui616@126.com (H.G.); nie@imau.edu.cn (R.N.); guot199608@163.com (T.G.); gaochang117@163.com (C.G.); m18353340167@126.com (J.M.); yuchaohu1994@163.com (Y.H.); wangwenwen2017@emails.imau.edu.cn (W.W.); liuna_dky@163.com (N.L.); xiaoping_an@163.com (X.A.); qijingwei@imau.edu.cn (J.Q.); 2 Key Laboratory of Smart Animal Husbandry, Universities of Inner Mongolia Autonomous Region, Hohhot 010018, China; 3 Inner Mongolia Herbivorous Livestock Feed Engineering Technology Research Center, Hohhot 010018, China

**Keywords:** complex plant extract, molecular mechanisms, gene expression regulation, lipid metabolism, multi-omics

## Abstract

Fatty acid contributes notably to meat nutrition value. A previous study investigated how complex plant extracts (CPE) can improve the fatty acid composition of ruminants, but the molecular mechanism remains unknown. This study aimed to examine the effect of dietary CPE supplementation on sheep growth performance and fatty acid composition in back fat (BF), combining lipidomics and transcriptomics analyses to explain the underlying mechanisms. Thirty-six female sheep, weighing 29.92 ± 2.52 kg and of a similar age (~4 months old), were randomly assigned into two groups: one received a basal diet (CTRL group), and the other received the same diet supplemented with 80 mg/kg of CPE (CPE group) for 75 days. The results revealed that the values of carcass fat content (GR) in the CPE group were significantly increased (*p* = 0.008), and the composition of fatty acid was changed. Lipidomic analysis indicated that CPE modulated lipid metabolism by regulating the contents of lipid molecules such as phosphatidylcholine (PC), fatty acyls (FAs), cardiolipin (CL), and triglyceride (TAG). After the addition of CPE, the lipid metabolism of BF was regulated mainly by regulating the glycerophospholipid metabolism, TNF signaling pathway, and cytokine–cytokine receptor interaction signaling pathway. These results revealed that changes in fatty acids were affected by the added CPE and corresponding molecular changes, which may provide new insights on the molecular level for applying CPE in sheep to improve fatty acid composition.

## Simple Summary

Complex plant extracts (CPE) have garnered significant attention in recent research owing to their impact on fatty acid compositions. Therefore, we employed CPE intervene in lambs and demonstrated that CPE regulated the lipid metabolism of back fat mainly by regulating the glycerophospholipid metabolism, TNF signaling pathway, and cytokine–cytokine receptor interaction signaling pathway. This regulation involved a series of related lipid molecules and genes. Novel insights on the molecular level for the application of CPEs in sheep to improve fatty acid composition are further provided.

## 1. Introduction

Mutton is widely produced and consumed globally due to its unique nutritional value and flavor characteristics. According to the National Bureau of Statistics, China’s mutton production has been increasing yearly, reaching 5.2 million tons in 2024 (http://www.stats.gov.cn, accessed on 2 December 2024). However, consumer demand for mutton is gradually shifting toward high-quality products. Various factors, including the breed, age, gender, and diet of the animal, profoundly influence the quality of mutton. Among these, dietary conditions are particularly crucial in determining meat quality [1].

Animal diet directly influences meat quality through its effects on fatty acid composition, antioxidant properties, and volatile content [2]. In recent years, the use of plant-derived active ingredients in animal feed to improve the fatty acid composition of mutton has garnered considerable attention. Plant polyphenols, which are abundant in plants, constitute a class of compounds known for their potent antioxidant properties. Research has shown that plant polyphenols and their derived phenolic active compounds can improve hepatic lipid metabolism in mice, thereby influencing fat deposition. Furthermore, these phenolic active compounds exhibit potentially distinct regulatory effects on fat deposition [3].

Recently, the natural phenolic compound eugenol has received significant attention. It is primarily sourced from the essential oils of plants such as cinnamon, clove, and basil. It has been found that eugenol significantly influences the aroma of beef by enhancing its flavor profile [4]. Lourenço et al. [5], using dual-flow continuous culture fermenters, demonstrated that adding eugenol can alter the composition of fatty acids by affecting the biohydrogenation process.

Another bioactive compound, cinnamaldehyde, extracted from cinnamon, possesses antibacterial and antiviral properties, effectively contributing to the healthy development of animals [6]. Previous research has shown that adding cinnamaldehyde to the diet can reduce the level of C18:0, while increasing the amounts of C18:1n9c, C18:2n6c, and C18:3n3 [7]. Additionally, cinnamaldehyde exhibits lipid-modulating effects, significantly decreasing serum triglyceride (TAG) levels while increasing plasma high-density lipoprotein cholesterol levels [8]. Furthermore, cinnamaldehyde has been shown to upregulate the gene expression of *PPARγ*, which is associated with lipid metabolism [9].

The main constituents of chili pepper oleoresin are capsaicinoids, which can effectively attenuate obesity and metabolic syndrome by favorably modulating lipid metabolism [10]. Capsaicinoids regulate lipid metabolism in animals by inhibiting lipoprotein lipase (*LPL*) and pancreatic lipase, stimulating lipolysis, and preventing adipocyte differentiation [11,12,13,14]. The addition of capsicum oleoresin to broiler feed can increase the fatty acid composition of breast and leg muscles [15]. Studies have shown that combining multiple natural plant extracts positively impacts animal growth performance and meat quality [16,17]. However, research on the effects of complex plant extracts (CPEs), which consist of eugenol, cinnamaldehyde, and capsicum oleoresin, on lipid deposition and metabolism in sheep remains limited.

Physiologically, communication occurs between fat and muscle tissues, and lipid accumulation in fat tissues can influence muscles, ultimately altering meat quality [18]. Given that the aforementioned plant extracts affect the body’s lipid metabolism, this study aims to investigate the effects of CPE on sheep growth characteristics, back fat (BF) deposition, and other relevant indicators. Furthermore, this study utilizes lipidomics and transcriptomics technologies to examine the effects of CPE on BF deposition and elucidate the mechanisms underlying the deposition process.

## 2. Materials and Methods

### 2.1. Animal and Experimental Design

The CPE (Pancosma XTRACT^®^ 7065) used in this study was provided by Pancosma (Shanghai, China) Feed Additives Co., Ltd. (Shanghai, China). The manufacturer guarantees that the active ingredient content of the microcapsule product is 5.5% cinnamaldehyde, 9.5% eugenol, and 3.5% capsicum oleoresin. A previous study showed that adding 80 mg/kg of CPE improved the production performance of ewes; hence, 80 mg/kg was selected as the dosage for this experiment [19].

Animal tests were conducted at the Hailiutu Experimental Base of Inner Mongolia Agricultural University. The experimental protocol was approved by the Inner Mongolia Agricultural University Research Ethics Committee (permission number [2020]065). The experiment lasted 75 days, including 15 days of pre-feeding and 60 days of the formal period. To eliminate sex differences, only female sheep were selected. Thirty-six sheep, approximately 4 months old with similar weight (29.92 ± 2.52 kg), were randomly divided into two groups. Each group contained six pens (six replicates), with three sheep raised in each pen. The experimental basal diet was formulated according to the meat sheep feeding standard (NY/T816-2004). The CTRL group was fed the basal diet without CPE, while the CPE group received the same diet supplemented with 80 mg/kg of CPE. The ingredients and nutrient levels of the basal diet are shown in Table 1.

Animals were fed twice daily (08:00 a.m. and 05:00 p.m.) to ensure a 10% refusal rate. Water was freely available throughout the experiment period. On the 61st day of the trial, one sheep was randomly selected from each pen, so a total of six sheep were slaughtered per group. Food was withheld for 12 h before slaughter, while water was restricted for 2 h. The animals were humanely slaughtered following Islamic ritual methods by severing the carotid artery and trachea. Approximately 15 cm of the BF tissue was collected, snap frozen in liquid nitrogen, and stored at −80 °C for fatty acid composition and lipidomic and transcriptomic analyses.

### 2.2. Carcass Traits

On day 61 of the trial period, six sheep were selected from each group for slaughter. Live weight was recorded prior to slaughter. Following slaughter, the head, hooves, and internal organs were removed, and the carcass was weighed after skin removal. The tissue thickness between the 12th and 13th ribs of the left carcass was measured using a vernier caliper at a distance of 11 cm from the spine’s central axis. This measurement was recorded as the carcass fat content (GR value). The muscle area of the longissimus dorsi between the 12th and 13th ribs of the left carcass was traced on sulfate paper and recorded as the loin eye area [20].

The main hot carcass weight, slaughter rate, and loin eye area were calculated using the following formulas:

Carcass weight (kg) = live weight before slaughter − weight of head, hooves, skin, hair, reproductive organs and surrounding fat, and internal organs (with kidneys and surrounding fat).

Slaughter rate (%) = (hot carcass weight ÷ live weight before slaughter) × 100.

Loin eye area (cm^2^) = eye muscle height × eye muscle width × 0.7.

### 2.3. Determination of Fatty Acid Composition

Sample pre-treatment: After slaughter, the tissue was weighed (55 mg), homogenized with 4 mL of n-hexane, and shaken at 50 °C for 30 min. Then, 3 mL of 0.4 mol/L KOH methanol solution was added and shaken at 50 °C for another 30 min. Next, 1 mL of H_2_O was added and mixed well. After phase separation, the upper layer was carefully collected, and an internal standard (methyl nonadecanoate, Sigma, N5377, Sigma-Aldrich, St. Louis, MO, USA) was added for gas chromatography–mass spectrometry (GC-MS) analysis.

Chromatographic analysis: The treated samples were analyzed using an Agilent chromatographic system (GC-MS 7890B-5977A, Agilent, Santa Clara, CA, USA) with the GC-MS external standard method. A DB-23 column (30 m × 320 μm × 0.25 μm) was used. The inlet and detector temperatures were set at 250 °C and 230 °C, respectively. Shunt injection was performed with a split ratio of 1:5. Helium was used as the carrier gas, and the sample injection volume was 1 μL. The heating procedure of the column box was as follows: (a) the initial temperature was maintained at 50 °C for 1 min; (b) then, the temperature was increased to 175 °C at a rate of 25 °C/min; (c) finally, the temperature was raised to 230 °C at a rate of 4 °C/min, and the whole heating procedure was held for 24.75 min.

### 2.4. Lipid Extraction, UHPLC-MS/MS Analysis, and Data Search

Lipid metabolite extraction and analysis followed the method described by Guo et al. [21]. Lipids were extracted using MTBE/methanol/water (10:3:2.5, *v*/*v*/*v*) with phase separation by centrifugation (1000× *g*, 10 min). The organic phase was dried under nitrogen, reconstituted in isopropanol, and analyzed by UHPLC-MS/MS (Q Exactive™ HF, Thermo Fisher, Waltham, MA, USA) with a C30 column (150 × 2.1 mm, 40 °C). Gradient elution (0.35 mL/min): 30% B (acetonitrile/isopropanol, 10:90) to 99% B over 16 min. Mobile phases contained 10 mM ammonium acetate and 0.1% formic acid. MS detection used a positive/negative switching mode (spray voltage 3 kV, *m*/*z* 114–1700) with a collision energy of 25–30 eV. Lipid raw data were then qualitatively and quantitatively analyzed, with data quality control applied before proceeding to bioinformatic analysis. Partial least squares discriminant analysis (PLS-DA) was performed using Python (version 3.5.0) to investigate group separation.

Differentially altered lipid molecules (DALs) between the CTRL and CPE groups were identified based on the following criteria: variable importance in projection (VIP) > 1.0, fold change (FC) > 1.2, or FC < 0.833 and *p* < 0.05 (calculated using an independent *t*-test). The VIP score was derived from the first principal component of the PLS-DA model, reflecting the contribution of each lipid molecule to group discrimination. The relative abundance of each lipid species was expressed as a percentage of total lipid molecules.

Pearson correlation analysis was performed to assess the relationship between individual DALs in each group using “SRplot”, an online platform for data analysis and visualization (accessed on 9 November 2023, https://www.bioinformatics.com.cn).

### 2.5. Transcriptome Analysis

RNA extraction and analysis followed the method described by Guo et al. [21]. Raw FASTQ data (raw reads) were initially processed using in-house Perl scripts. Clean data (clean reads) were obtained by removing adapter-containing, ploy-N-containing, and low-quality reads from the raw data. The clean data’s Q20, Q30, and GC contents were also determined. All subsequent analyses were conducted using high-quality and clean data.

Differential expression analysis between the CTRL and CPE groups was performed using the DESeq2 R package (version 1.20.0). Genes were considered differentially expressed genes (DEGs) if they met the threshold of |log2(FoldChange)| > 1 and *p* < 0.05. To assess the functional significance of the identified DEGs, an enrichment analysis of the Kyoto Encyclopedia of Genes and Genomes (KEGG) pathways and Gene Ontology (GO) terms was performed using the clusterProfiler R package (version 3.8.1). GO enrichment analysis was performed for the Biological Process (BP), Molecular Function (MF), and Cellular Component (CC) categories, while KEGG pathway analysis identified relevant biological pathways associated with differentially expressed genes.

### 2.6. Quantitative Real-Time PCR (qRT-PCR) Verification

The expression of candidate DEGs was verified using qRT-PCR as described by Guo et al. (2025) [21]. The mRNA sequences of sheep fatty acid desaturases 2 (*FADS2*), the elongase of very-long-chain fatty acids 7 (*ELOVL7*), and LPL were retrieved from the official National Center for Biotechnology Information (nih.gov).

Primers were designed using Primer Premier 5.0 software, and the primer sequences (Table 2) were synthesized by Sangon Biotech Co., Ltd. (Shanghai, China). The relative gene expression levels of the *FADS2*, *ELOVL7*, and *LPL* were calculated using the 2^−∆∆CT^ method [22].

### 2.7. Statistical Analysis and Data Visualization

After organizing and preprocessing the raw data using Excel 2021 software, data analyses were performed using the PROC GLM procedure in Statistical Analysis Software (SAS version 9.2; Cary, NC, USA) to assess treatment effects. Model assumptions, including the normality and homogeneity of variances, were checked using the Shapiro–Wilk and Levene’s tests, respectively.

Results were expressed by least square means with estimated standard error (SEM). Statistical significance was set at *p* < 0.05, with tendencies considered at 0.05 ≤ *p* < 0.10.

For data visualization, TBtools (v0.6673) [23] was used for bioinformatics analysis and graphical representation, GraphPad Prism v8 (GraphPad Software, San Diego, CA, USA) was employed for statistical plotting, and OmicShare [24] was utilized for pathway enrichment and network visualization.

## 3. Results

### 3.1. Effects of Supplementary CPE on Carcass Characteristics of Sheep

The effects of CPE supplementation on the carcass characteristics of sheep are shown in Table 3. After 60 days of feeding, no significant differences were observed in live weight, carcass weight, slaughter rate, or loin eye area between the CTRL and CPE groups (*p* > 0.05). However, the GR value of sheep in the CPE group was significantly elevated compared to that of the CTRL group (*p* < 0.05), indicating a greater fat content in the carcasses of the CPE group.

### 3.2. Fatty Acid Composition in BF

As shown in Table 4 and Table 5, 32 types of fatty acids were detected in both groups. Compared to the CTRL group, the contents of C16:0, C18:0, C18:2n6c, C18:3n6, C18:3n3, C20:4n6, and C22:1n9 in the BF of the CPE group were significantly decreased (*p* < 0.05). Additionally, the contents of C20:3n6 (*p* = 0.091), C21:0 (*p* = 0.084), and C24:0 (*p* = 0.085) showed a decreasing trend. Conversely, supplementation with CPE significantly increased the contents of C14:1, C16:1, and C18:1n9c in BF (*p* < 0.05).

As illustrated in Table 6, supplementation with CPE resulted in a significant decrease in saturated fatty acids (SFAs), polyunsaturated fatty acids (PUFAs), the PUFA/SFA ratio, and n-6 PUFA and n-3 PUFA levels in the BF of sheep in the CPE (*p* < 0.05). Conversely, the contents of unsaturated fatty acids (UFAs) and monounsaturated fatty acids (MUFAs) were significantly increased (*p* < 0.05).

### 3.3. CPE Changes the Lipid Profile Based on Lipidomic Analysis

To comprehensively discover lipid changes induced by dietary CPE supplementation, an untargeted lipidomic analysis was conducted by UHPLC-MS/MS in both negative and positive ion modes. The analysis aimed to quantify and compare lipid classes and individual lipid molecules between the CTRL and CPE groups.

**Figure 1:**
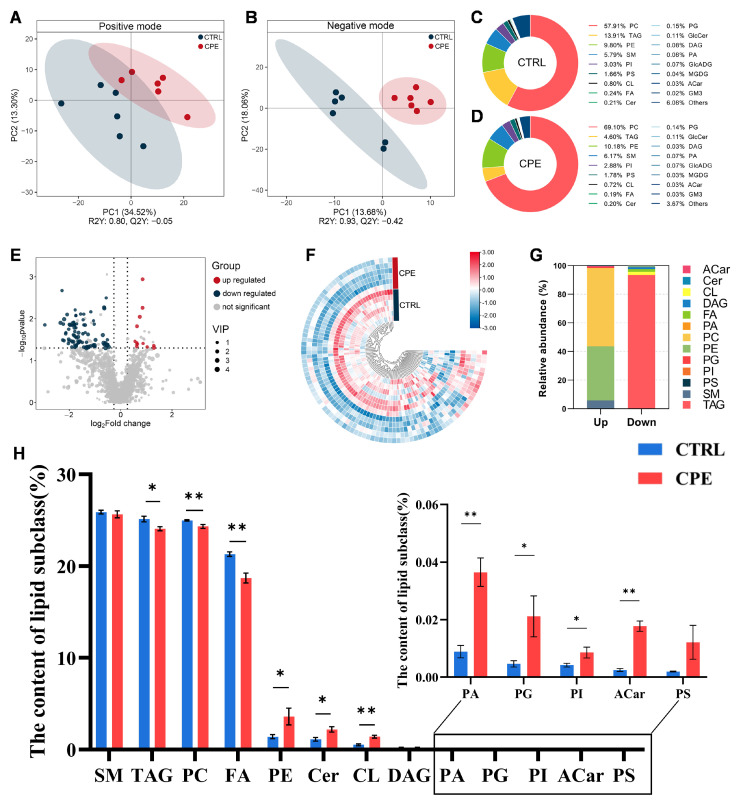
Effect of complex plant extract (CPE) supplementation on lipid characteristics of the back fat (BF) in sheep (n = 6). (**A**) Partial least squares discriminant analysis (PLS-DA) score plots in positive iron mode. (**B**) PLS-DA score plots in negative iron mode. (**C**,**D**) Percentage of lipid subclasses for the two groups. (**E**) The volcano plot shows all lipid molecules detected from the CTRL and CPE groups, with differentially upregulated and downregulated lipid molecules highlighted in red and blue, respectively. (**F**) The heatmap of the differentially altered lipid molecules (DALs) impacted by CPE, with blue for decreased and red for increased lipid levels. (**G**) Percentage of upregulated and downregulated lipid subclasses between the CTRL and CPE groups. (**H**) Histogram of the proportion of lipid molecules between the CTRL and CPE groups. Each column represents the percentage of that lipid molecule compared to all lipid molecules. Values are presented as means ± standard error (n = 6), * *p* < 0.05, and ** *p* < 0.01; CTRL, fed a basal diet; CPE, fed a diet supplemented with CPE added to the basal diet.

The PLS-DA model was employed to assess the degree of group separation and classification based on lipid profiles. The PLS-DA model showed clear discrimination between CTRL and CPE, indicating that CPE supplementation significantly impacted the lipid composition of BF. In positive ion mode, the PLS-DA model yielded scores of R^2^Y = 0.80 and Q^2^Y = −0.05, while in negative ion mode, the scores were R^2^Y = 0.93 and Q^2^Y = −0.42 (Figure 1A,B). Although Q^2^Y values were negative, suggesting limited predictive ability, R^2^Y > Q^2^Y in both cases indicates that the model is well established and still describes the data structure adequately.

In total, 1636 lipid molecules were identified and classified into 18 lipid subclasses (Figure 1C,D). Among these, the most abundant subclasses were phosphatidylcholine (PC), TAG, phosphatidylethanolamine (PE), and sphingomyelin (SM). Notably, CPE supplementation in diets significantly altered the lipid composition of BF. Specifically, CPE increased the relative abundance of PC from 57.91% to 69.10%, reduced TAG from 13.91% to 4.60%, and slightly increased PE (9.80% to 10.18%) and SM (5.79% to 6.17%) contents (Figure 1C,D), suggesting a shift in the lipid storage and membrane lipid composition

To further examine the effects of CPE on the lipid profile of BF, DALs were screened according to the previously mentioned criteria. A total of 97 DALs were identified between the CTRL and CPE groups, including 12 upregulated and 85 downregulated lipid species, as presented in Figure 1E,F (the details of DALs are presented in Table S1).

As shown in Figure 1G,H, the 97 DALs were distributed across 13 lipid subclasses, demonstrating significant compositional shifts in lipid composition. The downregulated DALs in BF mainly belong to PC and fatty acyls (FAs), suggesting suppressed storage or membrane lipid synthesis pathways. In contrast, the upregulated DALs were primarily associated with cardiolipins (CLs), phosphatidic acid (PA), and acyl-carnitine (Acar), which are involved in mitochondrial function, lipid signaling, and fatty acid oxidation, respectively. These results suggest that CPE supplementation may enhance lipid utilization and mitochondrial activity while reducing lipid accumulation in sheep BF.

### 3.4. CPE Changes the Lipid Profile Based on Transcriptomic Analysis

**Figure 2:**
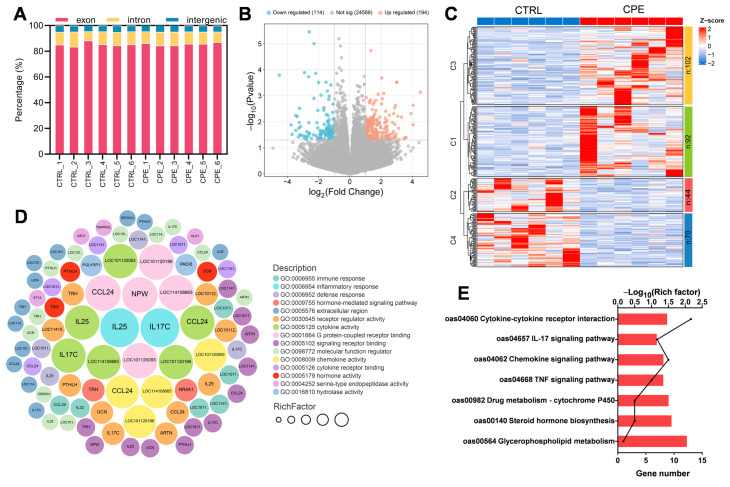
Back fat (BF) tissue transcriptomic profiles of the control (CTRL) and complex plant extract (CPE) groups. (**A**) Statistical diagram for the distribution of reads mapped to the reference genome. (**B**) Significantly up- and downregulated genes are represented as “orange” and “blue” points in the volcano plot, respectively. (**C**) Heatmap of differentially expressed genes (DEGs) in the comparison of CTRL vs. CPE. (**D**) Gene Ontology (GO) enrichment analysis of DEGs between the CTRL and CPE groups. (**E**) Kyoto Encyclopedia of Genes and Genomes (KEGG) enrichment result plots of CTRL vs. CPE comparison groups.

To explore the gene expression profile of BFs after CPE supplementation, RNA sequencing was performed on both the CTRL and CPE groups (n = 6). This process generated 81.69 GB of raw data across 12 libraries, with at least 91.66% of the reads achieving quality scores of Q30 or higher (Table S2). Following the removal of low-quality reads and adapters, an average of 91.08% of clean reads were successfully and uniquely mapped to the reference genome (Table S3). In the annotation files for uniquely aligned sequences, 85.48%, 9.72%, and 4.80% of reads aligned to exon, intron, and intergenic regions, respectively, per sample (Figure 2A), ensuring high reliability for downstream analyses. A total of 308 DEGs were identified, of which 114 were downregulated and 194 were upregulated in CPE compared with CTRL (Figure 2B,C).

**Figure 3:**
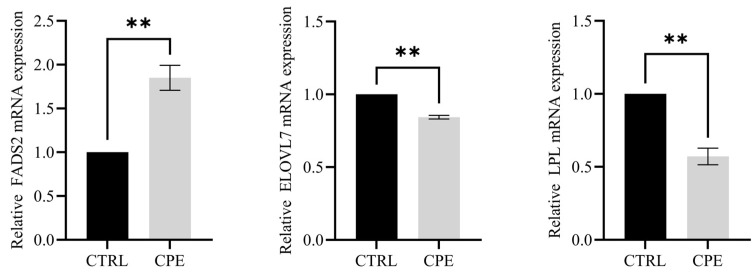
Histogram of relative gene expression in BF. Values are presented as means ± standard error (n = 6), and ** *p* < 0.01; CTRL, fed a basal diet; CPE, fed a diet supplemented with CPE added to the basal diet.

To further investigate the biological functions, DEGs were evaluated by GO functional enrichment analysis. In the CTRL vs. CPE comparison, DEGs were enriched in cytokine activity, G protein-coupled receptor binding, chemokine activity, and inflammatory response (Figure 2D). As shown in Figure 2E, KEGG pathway enrichment analysis indicated that cytokine–cytokine receptor interaction and glycerophospholipid metabolism were directly related to lipid metabolism in LDM after CPE supplementation. Additionally, altered genes were significantly enriched in the IL-17 and TNF signaling pathways (Figure 2E). These results suggest that CPE supplementation may exert regulatory effects on lipid deposition in BF through inflammation-related signaling pathways. In addition, we examined the expression levels of three genes related to lipid metabolism, including *ELOVL7*, *LPL*, and *FADS2*. The results show that the expressions of these genes showed expression trends consistent with the transcriptome data (Figure 3).

## 4. Discussion

The GR value serves as a crucial indicator, reflecting the content of carcass adipose tissue, wherein higher values denote greater fat deposition. A previous study suggested that supplementing plant extracts in diets can enhance lipid accumulation in livestock [25]. In this study, the addition of CPE did not affect the live weight before slaughter, hot carcass weight, slaughter rate, and lion eye area but significantly increased GR values, suggesting that CPE promotes adipose tissue deposition in sheep.

The composition and content of FAs in adipose tissue are closely associated with its nutritional value. SFAs have long been considered detrimental to human health due to their association with an increased risk of cardiovascular diseases, raising concerns regarding their excessive intake. However, our results demonstrate a significant reduction in the SFA content following CPE supplementation. This reduction in SFA levels may be attributed to the unique properties of CPE, which potentially inhibit the synthesis of SFAs.

Conversely, higher levels of UFAs and lower levels of SFAs have been linked to improved lipid profiles, particularly through the reduction in LDL-C, thereby lowering the risk of cardiovascular diseases [26]. The conversion of SFA to UFA necessitates a series of desaturase-catalyzed reactions, primarily yielding beneficial MUFAs such as C16:1 and C18:1n9c [27]. In this study, the addition of CPE significantly increased the MUFA content, indicating that CPE supplementation not only promotes adipose tissue deposition but also improves fat deposition. The observed decrease in SFAs and increase in MUFAs suggest that CPE enhances the nutritional value of meat products by shifting the lipid profile toward a more favorable composition.

Furthermore, C18:1n9c, the most abundant and beneficial MUFA in ruminants, produces distinct flavor compounds during cooking and has been shown to exhibit protective effects against cardiovascular diseases [28]. In this study, the addition of CPE resulted in a significant increase in the C18:1n9c content, thereby enhancing both the sensory quality and health value of sheep meat.

Interestingly, PUFA levels, the PUFA/SFA ratio, n-6 PUFA, and n-3 PUFA were significantly reduced following CPE supplementation. Research suggests that mammals lack the Δ-15 desaturase enzymes, which are necessary for the endogenous synthesis of PUFAs, making dietary intake the primary source [29]. Therefore, the reduction in PUFA content in the BF of sheep fed with CPE may be attributed to the phenolic compounds’ inhibitory effect on the enzyme activity required for PUFA synthesis.

Lipid peroxidation is a major factor contributing to meat quality deterioration, affecting color stability and nutritional value, and may lead to the formation of potentially toxic compounds. Studies indicate lipid oxidation strongly correlates with lipid molecules, particularly TAG, which belongs to the GL and GP classes [30,31]. Since fatty acid composition plays a critical role in lipid oxidation, a lower PUFA content is often associated with enhanced oxidative stability of meat. Specifically, n-3 PUFA has been linked to increased lipid oxidation and TAG content reduction [32,33].

Lipidomics analysis revealed a decrease in TAG content in sheep BF following CPE supplementation, along with a significant reduction in n-3 PUFA content. This suggests that CPE supplementation may decrease TAG content by lowering n-3 PUFA levels, thereby mitigating lipid oxidation and enhancing meat quality. Additionally, elevated free fatty acid levels in the blood have been associated with increased TAG accumulation in the liver and muscle. This can inhibit fat synthesis while promoting TAG breakdown, ultimately leading to reduced de novo SFA synthesis [34]. Consistent with this, our study found a significant decrease in both TAG and SFA content following CPE supplementation, further supporting the role of CPE in modifying lipid metabolism and enhancing the nutritional quality of sheep meat.

Similarly, a study by Liu [35] demonstrated that adding phenol-rich black artemisia to cashmere goat diets led to a significant reduction in TAG content in the longissimus dorsi muscle, thereby enhancing the nutritional quality of mutton. In this experiment, the lipid subclasses PE, PI, PC, PG, CL, and PA were significantly regulated after adding CPE. These subclasses are associated with GP, which plays a key role in lipid oxidation. Among them, PC and PE are the most abundant. Research indicates that the composition of GP is closely linked to the flavor profile of meat products [36].

PC, a major structural component of cell membranes, consists of glycerol, two fatty acid chains, and one phosphate ester group, constituting 45% to 55% of the total phospholipid in mammalian cells and organelles. PC metabolism is closely intertwined with fatty acid metabolism, where UFAs within PC are highly susceptible to oxidation, influencing both fatty acid synthesis and breakdown. Consistent with our findings, CPE supplementation led to a significant reduction in PC content and a notable increase in UFA content, suggesting that PC metabolism could modulate the fatty acid composition of BF by regulating UFA levels.

SFAs typically found in PC include C18:0 and C16:0. In contrast, UFAs include C18:1, C18:2, C18:3, and C20:4. The findings from this study showed a significant decrease in PC content and fatty acids within PC in BF following CPE supplementation. Similarly, PE is involved in glycine, serine, threonine, and glycerophospholipids metabolic pathways [37]. Li et al. [38] demonstrated that shallot extract can enhance meat quality by modulating PE content. Consistent with our study’s findings, adding CPE upregulates PE levels and subsequently modulates the fatty acid composition in BF.

Fatty acid desaturases (FADS) are crucial in PUFA biosynthesis, catalyzing dehydrogenation reactions that introduce double bonds at specific acyl chain positions. FADS2 and FADS6 function as Δ-6 desaturases, which are responsible for inserting double bonds at Δ-6 (Δ-12) positions. Through transcriptomics analysis, Wang et al. [39] identified FADS2 as a candidate gene in lipid metabolism, modulating fatty acid unsaturation. Consistent with our findings, dietary CPE supplementation significantly upregulated FADS2 gene expression, resulting in a decrease in SFA content and an increased UFA content, indicating FADS2’s regulatory role in the fatty acid composition. Li et al. [40] found that supplementing Hu sheep’s diet with grape seed tannin extract, a phenolic substance, upregulated the expression of the FADS2 gene, leading to a significant reduction in C18:0 content.

The elongase of the very-long-chain fatty acid (ELOVLs) family is another key regulator of lipid metabolism and has garnered considerable interest. ELOVLs, primarily in the endoplasmic reticulum, are crucial rate-limiting enzymes in long-chain PUFA synthesis [41]. Specifically, ELOVL7 catalyzes the elongation of both SFAs and PUFAs [42,43]. In line with this study, dietary CPE supplementation notably downregulated ELOVL7 gene expression, resulting in a reduction in SFA and PUFA contents. Previous research indicates that the reduction in C18:1n9c content results from ELOVL7 overexpression, with C18:1n9c content exhibiting a negative correlation with ELOVL7 expression, affirming ELOVL7’s involvement in fatty acid metabolism [43]. Consistently, CPE supplementation downregulated ELOVL7 gene expression while significantly increasing C18:1n9c content, further supporting its role in fatty acid metabolism.

LPL serves as a crucial rate-limiting enzyme in the hydrolysis of TAG into glycerol and free fatty acids [44]. TAG, transported in the bloodstream via very-low-density lipoprotein (VLDL), undergoes hydrolysis by LPL, generating free fatty acids that are either stored in adipose tissue or used for energy metabolism. The expression of LPL is subject to regulation by hormones, fasting, supplementary feeding, and dietary factors [45]. Tian Jia [46] discovered, in their study, a significant negative correlation between LPL gene expression and UFA content in the triceps brachii muscle of Altai sheep. Similarly, Shimomura et al. [47] posited that elevated UFA levels could disrupt the transcription of lipase genes, consequently impeding body metabolism and downregulating LPL gene expression. In agreement with these findings, the UFA content significantly increased while LPL gene expression decreased after dietary supplementation with CPE, suggesting that CPE might augment UFA content by suppressing LPL gene expression.

Notably, capsaicin exhibited an inhibitory effect on LPL activity, indicating a potential regulatory role of chili oleoresin present in CPE on sheep BF lipid metabolism via LPL activity modulation. Moreover, the reduction in TAG content observed in this study suggests that the LPL-mediated hydrolysis of TAG was suppressed, leading to lower free fatty acid availability and decreased TAG accumulation in adipose tissue [48]. Consequently, the reduced expression of the LPL gene leads to decreased free fatty acids produced by lipid metabolism, subsequently lowering TAG accumulation in adipose tissue. In conclusion, *FADS2*, *ELOVL7*, and *LPL* are potential key genes regulating fatty acid composition in BF.

Earlier research suggested that fatty acids deposited in muscle tissue primarily originate from the bloodstream. However, subsequent studies have demonstrated that amino acids regulate bodily fatty acid metabolism. Hayashi [49] observed a significant enhancement in lymph lipid transport within the rat intestine when amino acids were supplemented in the diet, indicating an interplay between amino acid and fatty acid metabolism. A study confirmed that the phenols in the CPE utilized in this study possess lipid peroxidation-reducing properties [27]. A metabolomics study by Jintao [35] demonstrated that supplementing the diet of Artemisia nigrosalis with phenolic substances could mitigate blood lipid peroxidation, facilitate lipid decomposition, lower blood TAG content, and decrease blood C16:0 and C18:0 levels via the arginine and proline metabolic pathways. Moreover, a reduction in the deposition of SFAs, such as C16:0 and C18:0, in muscle was observed, improving meat quality.

Correspondingly, the dietary supplementation of CPE led to a significant reduction in C16:0, C18:0, and SFA levels in BF, along with a downward trend in TAG content. KEGG pathway enrichment analysis identified several DEGs significantly associated with the arginine and proline metabolic pathways. These findings suggest that CPE might mitigate lipid peroxidation by these metabolic pathways, consequently enhancing sheep BF’s fatty acid composition. The synthesis of fatty acids requires considerable energy, including n-3 PUFA. Research indicates that C16:1 can enhance energy availability for white fat metabolism by stimulating lipolysis, glucose uptake, and utilization [50,51,52]. With CPE supplementation, the C16:1 content in sheep BF increased, whereas n-3 PUFA levels decreased notably. This phenomenon might be attributed to a reduction in TAG content in BF, which typically releases ample ATP upon decomposition.

Furthermore, most DEGs significantly associated with the adenosine triphosphate pathway showed a decrease, suggesting a reduced energy demand for n-3 PUFA synthesis. Moreover, KEGG enrichment analysis revealed significant enrichment of pathways such as the IL-17 and TNF signaling pathways, which are associated with inflammation.

Lipid oxidation regulates various physiological processes, including inflammation, immune defense, and metabolic stress [53]. Putman et al. [54] reported a strong association between lipid oxidation and an increased incidence of postpartum inflammatory diseases in cows. In the present study, dietary supplementation with CPE markedly decreased n-3 PUFA and TAG levels in BF, thereby attenuating the lipid oxidation process. Hence, this suggests that the regulatory impact of CPE on lipid metabolism in adipose tissue may be linked to modulating the inflammatory response. However, further research is needed to elucidate the underlying mechanism.

## 5. Conclusions

This study aimed to evaluate the effects of dietary supplementation with CPE on the growth performance and fatty acid composition in sheep’s BF. The experimental data demonstrate that the live weight, carcass weight, and other growth-related parameters showed no significant differences, whereas the GR value was significantly elevated after CPE supplementation. Regarding fatty acid composition, CPE significantly reduces SFA levels while increasing UFA and MUFA levels. The modulation of lipid metabolism in BF by CPE primarily involves the alteration in glycerolipid and glycerophospholipid metabolism. Transcriptomic analysis revealed that cytokine–cytokine receptor interaction and the TNF signaling pathway may regulate CPE-mediated lipid metabolism.

In conclusion, this study suggests that CPE can regulate lipid metabolism and provides a novel perspective on improving fatty acid composition in sheep at the molecular level. However, further studies are needed to clarify the precise mechanism underlying CPE’s influence on lipid metabolism.

## Figures and Tables

**Table 1:** Ingredients and chemical composition of the basal diet fed to the sheep ^1^.

Items	Content, %
Ingredients	
Maize grain	35.10
Soybean meal (43% crude protein)	5.00
Cottonseed meal	5.00
Corn germ meal	23.00
Sunflower seed shells	13.00
Rice bran meal	12.00
Limestone	1.50
Salt	0.70
Vitamin–mineral premix ^1^	2.00
Dicalcium phosphate	0.70
Bentonite	2.00
Total	100.00
Chemical composition, %	
Metabolizable energy, MJ/kg ^2^	9.36
Crude protein, %	14.39
Neutral detergent fiber, %	33.99
Acid detergent fiber, %	12.97
Ash, %	10.01
Calcium, %	1.01
Phosphorus, %	0.69

^1^ The premix provided the following per kilogram: vitamin A of 350,000 IU, vitamin D3 of 93,750 IU, vitamin E of 938 mg, vitamin K3 of 63 mg, vitamin B1 of 62 mg, vitamin B2 of 188 mg, nicotinamide of 750 mg, pantothenic acid of 500 mg, vitamin B6 of 62 mg, biotin of 3.7 mg, folic acid of 38 mg, Se of 18 mg, Zinc of 3000 mg, iodine of 23 mg, Co of 30 mg, Mn of 2500 mg, Fe of 3240 mg, and Cu of 500 mg. ^2^ Metabolizable energy was calculated, and other nutrients were measured.

**Table 2:** Primer sequences used for quantitative qRT-PCR.

qRT—PCR Target ^1^	Sequences of Primers (5′–3′)	Product Size/bp
*FADS2*	F: TCCGCTGGGAGGAGATT	131
	R: GGCGTAGTGCCCGATG	
*ELOVL7*	F: AAGAGGCTGGTGGGCTACA	127
	R: GGCTACCGAAGATGAGGGA	
*LPL*	F: AAAGAGAAAAGTTACAGTCAGTCAT	139
	R: AAATAAATGTTCACTCACTCTTG	

^1^ qRT-PCR: Quantitative real-time PCR; *FADS2*: fatty acid desaturases 2; *ELOVL7*: elongase of very-long-chain fatty acids 7; *LPL*, lipoprotein lipase.

**Table 3:** Effects of CPE supplementation on carcass characteristics of sheep (n = 6).

Items	Group ^1^		SEM ^3^	*p*-Value
	CTRL	CPE		
Live weight before slaughter (kg)	41.24	43.10	0.57	0.454
Hot carcass weight (kg)	21.87	22.36	0.25	0.580
Slaughter rate (%)	53.33	53.35	0.01	0.985
Loin eye area (cm^2^)	17.88	17.89	0.48	0.460
GR value (mm) ^2^	15.95	18.68	1.37	0.008

^1^ The control (CTRL) group was fed the basal diet. The complex plant extract (CPE) group was fed the basal diet supplemented with 80 mg/kg of CPE. ^2^ GR value, a measurement to estimate carcass fat content. ^3^ SEM, standard error of the mean.

**Table 4:** Effects of CPE supplementation on polyunsaturated fatty acid (PUFA) composition (%) of BF in sheep (n = 6).

Items	Groups ^1^		SEM ^2^	*p*-Value
	CTRL	CPE		
n3 PUFA				
C18:3n3	0.236	0.165	0.016	0.019
C20:3n3	0.031	0.028	0.002	0.376
C20:5n3	0.012	0.014	0.001	0.418
C22:6n3	0.019	0.02	0.001	0.962
n6 PUFA				
C18:2n6c	3.673	2.521	0.222	0.003
C18:3n6	0.036	0.029	0.002	0.006
C20:2n6	0.052	0.048	0.003	0.548
C20:3n6	0.011	0.009	0.000	0.091
C20:4n6	0.128	0.105	0.004	0.001

^1^ The control (CTRL) group was fed the basal diet. The complex plant extract (CPE) group was fed the basal diet supplemented with 80 mg/kg of CPE. ^2^ SEM, standard error of the mean.

**Table 5:** Effects of CPE supplementation on monounsaturated and saturated fatty acid profile compositions (%) of BF in sheep (n = 6).

Items	Groups ^1^		SEM ^2^	*p*-Value
	CTRL	CPE		
C6:0	0.002	0.002	0.000	0.716
C8:0	0.011	0.01	0.001	0.711
C10:0	0.170	0.155	0.009	0.424
C11:0	0.010	0.009	0.001	0.526
C12:0	0.155	0.125	0.017	0.409
C13:0	0.028	0.025	0.003	0.522
C14:0	2.741	2.844	0.152	0.753
C14:1	0.364	0.553	0.049	0.046
C15:0	0.910	0.803	0.072	0.485
C15:1	0.132	0.146	0.031	0.834
C16:0	25.802	23.289	0.445	0.001
C16:1	2.120	2.670	0.091	<0.0001
C17:0	3.357	3.412	0.279	0.927
C17:1	1.263	1.424	0.122	0.537
C18:0	14.595	11.474	0.570	0.001
C18:1n9c	43.991	49.703	1.030	0.001
C20:0	0.089	0.084	0.006	0.693
C20:1	0.093	0.100	0.007	0.633
C21:0	0.012	0.009	0.001	0.084
C22:0	0.009	0.006	0.001	0.107
C22:1n9	0.009	0.006	0.001	0.05
C23:0	0.010	0.010	0.001	0.872
C24:0	0.003	0.003	0.000	0.085

^1^ The control (CTRL) group was fed the basal diet. The complex plant extract (CPE) group was fed the basal diet supplemented with 80 mg/kg of CPE. ^2^ SEM, standard error of the mean.

**Table 6:** Effects of CPE on saturated fatty acid (SFA), unsaturated fatty acid (UFA), polyunsaturated fatty acids (PUFAs), and monounsaturated fatty acids (MUFAs) of BF (%) in sheep (n = 6).

Items	Groups ^1^		SEM	*p*-Value
	CTRL	CPE		
SFA	47.903	42.259	1.019	0.001
UFA	52.173	57.541	1.093	0.006
PUFA	4.200	2.938	0.239	0.002
MUFA	47.973	54.603	1.202	0.001
PUFA/SFA	0.088	0.070	0.004	0.019
n-6 PUFA	3.869	2.683	0.014	0.002
n-3 PUFA	0.279	0.206	0.227	0.000
n-6/n-3 PUFA	13.856	12.997	0.761	0.959

^1^ The control (CTRL) group was fed the basal diet. The complex plant extract (CPE) group was fed the basal diet supplemented with 80 mg/kg of CPE.

## Data Availability

The data presented in this study are openly available in the Sequence Read Archive (SRA)of NCBI, reference number [PRJNA1224501].

## Supplementary Materials

The following supporting information can be downloaded at: https://www.mdpi.com/article/10.3390/ani15111645/s1, Table S1: Detailed information on differential lipids; Table S2: Data quality control of sequencing; Table S3: Mapping rate of sequence libraries.

## Abbreviations

The following abbreviations are used in this manuscript:

FADS2	Fatty acid desaturases 2
FC	Fold change
GC-MS	Gas chromatography–mass spectrometry
GO	Gene Ontology
GR value	Carcass fat content
KEGG	Kyoto Encyclopedia of Genes and Genomes
LPL	Lipoprotein lipase
MF	Molecular function
MUFAs	Monounsaturated fatty acids
PA	Phosphatidic acid
PC	Phosphatidylcholine
PE	Phosphatidylethanolamine
PLS-DA	Partial least squares discriminant analysis
PUFAs	Polyunsaturated fatty acids
SEM	Standard error
SFAs	Saturated fatty acids
SM	Sphingomyelin
TAG	Triglyceride
UFAs	Unsaturated fatty acids
VIP	Variable importance in projection
VLDL	Very-low-density lipoprotein
