# Supplementary material for: Lipidomics and Transcriptomics Analyses Reveal Dietary Complex Plant Extracts Improve Lipid Composition of Back Fat in Sheep
Source: Animals (Basel). 2025 Jun 3;15(11):1645. doi: 10.3390/ani15111645 (PMC12153842; doi:10.3390/ani15111645)
Supplement: Supplementary file 1 [file animals-15-01645-s001.zip › Supplementary material.pdf]

Table S2. Data quality control of sequencing

Sample[1]	Raw reads	Raw bases (G)	Clean reads	Clean bases (G)	Q20[2] (%)	Q30[2] (%)	GC content (%)
CTRL_1	45469154	6.82	43915384	6.59	97.17	92.44	51.43
CTRL_2	47289150	7.09	45714216	6.86	96.97	91.95	50.67
CTRL_3	42489544	6.37	41172550	6.18	97.24	92.61	51.48
CTRL_4	46193214	6.93	45121142	6.77	97.18	92.47	51.59
CTRL_5	45595628	6.84	44236766	6.64	96.85	91.66	50.82
CTRL_6	47051066	7.06	45947756	6.89	97.07	92.18	51.93
CPE_1	42342758	6.35	40778202	6.12	97.09	92.31	52.56
CPE_2	47314152	7.10	45601632	6.84	97.22	92.50	51.45
CPE_3	43805030	6.57	42033364	6.31	97.07	92.28	51.86
CPE_4	45472690	6.82	44666980	6.70	97.16	92.40	50.82
CPE_5	44992410	6.75	43742452	6.56	97.41	92.97	51.61
CPE_6	46606720	6.99	45436444	6.82	97.13	92.35	51.72

[1]12 samples from both control (CTRL) and composite plant extracts (CPE) groups.

[2]Represents the proportion of 1 incorrectly identified base per 1000 bases, with a correct recognition rate of 99.9%.

Table S3. Mapping rate of sequence libraries

Sample[1]	total_reads	total_map	unique_map	multi_map	proper_map
CTRL_1	43915384	41747707(95.06%)	39933743(90.93%)	1813964(4.13%)	20008888(45.56%)
CTRL_2	45714216	43396102(94.93%)	41467234(90.71%)	1928868(4.22%)	20828399(45.56%)
CTRL_3	41172550	39269513(95.38%)	37386902(90.81%)	1882611(4.57%)	18741017(45.52%)
CTRL_4	45121142	43124332(95.57%)	41441358(91.84%)	1682974(3.73%)	20770704(46.03%)
CTRL_5	44236766	41923563(94.77%)	39824018(90.02%)	2099545(4.75%)	20033385(45.29%)
CTRL_6	45947756	43889141(95.52%)	42205259(91.85%)	1683882(3.66%)	21182953(46.1%)
CPE_1	40778202	38643901(94.77%)	37149835(91.1%)	1494066(3.66%)	18632610(45.69%)
CPE_2	45601632	43317052(94.99%)	41345795(90.67%)	1971257(4.32%)	20735620(45.47%)
CPE_3	42033364	39868565(94.85%)	38291204(91.1%)	1577361(3.75%)	19199523(45.68%)
CPE_4	44666980	42802476(95.83%)	40945055(91.67%)	1857421(4.16%)	20535345(45.97%)
CPE_5	43742452	41808165(95.58%)	39934879(91.3%)	1873286(4.28%)	19984860(45.69%)
CPE_6	45436444	43341021(95.39%)	41320463(90.94%)	2020558(4.45%)	20723666(45.61%)

[1]CTRL, fed a basal diet. CPE, fed a diet supplemented with CPE added to the basal diet.
